# Legal access to medications: a threat to Brazil’s public health system?

**DOI:** 10.1186/s12913-017-2430-x

**Published:** 2017-07-19

**Authors:** Ana Luiza Chieffi, Rita De Cassia Barata Barradas, Moisés Golbaum

**Affiliations:** 1 Preventive Medicine Department, Faculty of Medicine of University of São Paulo, Av. Dr. Arnaldo, 455-2nd floor - Room 2168, Cerqueira César, São Paulo, 01246-903 Brazil; 2 Santa Casa de São Paulo School of Medical Sciences, Researcher 1C of National Council for Scientific and Technological Development-CNPq, São Paulo, Brazil; 3 Preventive Medicine Department, Faculty of Medicine of University of São Paulo, Researcher 1C of National Council for Scientific and Technological Development-CNPq, São Paulo, Brazil

**Keywords:** Judicialization of health, Universal Health System, Right to health, Pharmaceutical assistance

## Abstract

**Background:**

In Brazil, health is fundamental human right guaranteed by the Constitution of 1988, which created the Brazilian Universal Health System (Sistema Único de Saúde - SUS). The SUS provides medications for outpatient care via policy of pharmaceutical assistance (PA) programmes. Despite the advances in PA policies which include the improvement in access to medications, there has been a significant increase in lawsuits related to health products and services. This study aimed to characterize the medication processes filed between 2010 and 2014 against the Secretary of State for Health of São Paulo (State Health Department of São Paulo - SES/SP), in Brazil, following PA policies.

**Methods:**

This descriptive study used secondary data on medication lawsuits filed against the SES/SP between 2010 and 2014. The data source was the S-Codes computerized system.

**Results:**

In the period evaluated, the number of lawsuits filed concerning health-related products increased approximately 63%; requests for medications were predominant. Approximately 30% of the medications involved in court proceedings were supplied via PA programmes. With regard to medications supplied via specialized component, 81.3% were prescribed in disagreement with the protocols published by the Ministry of Health. Insulin glargine was the most requested medication (6.3%), followed by insulin aspart (3.3%). Because there is no scientific evidence that either of these medicines is superior for the treatment of diabetes, neither of them has been incorporated into the SUS by the National Commission for Technology Incorporation. The judicial data showed that most of the lawsuits involved normal proceedings (i.e., individual demands), were filed by private lawyers, and named the State of São Paulo as the sole defendant, demonstrating the individual nature of these claims.

The data indicate inequality in the distribution between the number of cases and lawyers and the number of lawsuits and prescribers, evidencing the concentration of lawyers and physicians in filing lawsuits.

**Conclusion:**

The judicialization of health in the State of São Paulo with the characteristics presented herein is a threat to the SUS.

## Background

In Brazil, health is a citizen’s fundamental right guaranteed by the Constitution of 1988, which created the Brazilian Universal Health System (Sistema Único de Saúde - SUS) [1]. The SUS is composed of a set of health actions and services. It is a regionalized and hierarchical network whose constitutional principles include universality, comprehensiveness of care, and equity. Brazil’s three governmental spheres (federal, state, and municipal) share the responsibility of guaranteeing the right to health [2].

Access to medications constitutes the guiding principle of the public policies that have been established in the area of pharmaceutical assistance (PA): these products are used for basic therapeutic interventions and directly impact the development of health actions. According to the World Health Organization (WHO), the primary objectives of medicines policies are access to and the rational use of high-quality essential medicines [3].

The SUS offers free medications for outpatient care via PA programmes. Treatments are provided for the most common diseases via primary health care units (PHCU) via the basic PA component; medications and supplies are provided for the prevention, diagnosis, treatment, and control of diseases and endemic diseases via the strategic PA component; and medications whose care strategies have been defined in clinical protocols and therapeutic guidelines (CPTG) published by the Ministry of Health are provided via the specialized PA component [4, 5].

The basic component dispenses medicines for the treatment of, inter alia, hypertension and other cardiovascular diseases, childhood infections and respiratory illnesses, infant dehydration, intestinal parasites, and mental disorders. The strategic component dispenses medicines for the treatment of nutritional disorders, bleeding disorders, cholera, nicotine addiction, Chagas disease, dengue, graft versus host disease, schistosomiasis, filariasis, leprosy, HIV/AIDS, influenza, leishmaniasis, systemic lupus erythematosus, malaria, meningitis, systemic mycoses, multiple myeloma, human rabies, trachoma, and tuberculosis. The specialized component provides medicines to treat 78 diseases, as described in Appendix 1 [4].

To add to the medicines available via PA programmes, the Ministry of Health established the Popular Pharmacy Program in 2004. This programme provides medicines either free of charge or at-cost for the treatment of hypertension, diabetes, asthma, dyslipidaemia, rhinitis, Parkinson’s disease, osteoporosis, and glaucoma and contraceptives [5].

In oncology, patient care involves the use of physician-provided protocols and therapeutic guidelines, the supply of medications, and the comprehensive treatment of patients with cancer via promotion, prevention, early detection, adequate treatment, and palliative care [6].

Brazil’s states and municipalities use the items of the programmes described above and standardize the medications that they provide, thereby increasing the number of available medicines.

Despite advances in the PA policy and increased access to medication, the number of medications and health treatments obtained through resort to the legal system has increased [7]. The judicialization of health involves court decisions that require the government to provide health products and services based on the right to health, as defined by the Federal Constitution of 1988. The Brazilian judicial system has tended to accept individual demands which claim the supply of medicines, supplies and health treatments by SUS, considering that the right to health guarantees citizens the right to receive those products whenever necessary, medically indicated, and the refusal to supply by the public authorities [8].

Medicines lawsuits are considered a major challenge to the SUS. These lawsuits have the positive effect of ensuring access to medications, particularly in cases of shortage or a delay in the inclusion of medications into the SUS. Nevertheless, they can also have adverse effects in that they can compromise the principles of SUS, generating difficulties in PA management and promoting the careless use of medications, all of which subject applicants’ health to unnecessary risks [9].

Health managers have attempted to respond to lawsuits, which cause serious administrative and financial problems because of the exponential increase in the number of claims made and the impossibility of forecasting the costs associated with them [7]. In contrast, the positive aspects of the judicialization of health should be considered. The judicialization of health encourages the provision of solutions by agents of the public health sector to compensate for possible failures and malfunctions in SUS regulations, avoid further litigation, and preserve the principles and guidelines of SUS. It is also possible to identify a positive correlation between access to justice and the promotion of the right to health based on the assumption that judicialization is not always a problem to solve or a failure of the judicial system [10].

The lawsuits against the State Health Secretary of São Paulo (Secretaria de Estado de Saúde de São Paulo-SES/SP) began in the early 1990s, and since that time, they have increased annually. In recent years, lawsuits have become routine. For many years, the increased number of lawsuits against the public network to request medications and health care services has been attributed to the SUS’s lack of equity [7]. However, studies have shown that this phenomenon brought more inequality to the system [11] by focusing on richer patients, who have increased access to health care and the court system [7].

The results of studies on the judicialization of health in some Brazilian states have indicated that most lawsuits are individual [8], filed by private lawyers to request medicines that are not covered by the SUS’s PA programmes. In several Brazilian states, there is a strong correlation between high socioeconomic status and the number of lawsuits filed [7, 12, 13]. Currently, the steady increase in the number of lawsuits is attributed to poor management of PA [11], delays in the supply of medications, inaction, and insufficient incorporation of new technologies into the SUS [14]; medicines are the emphasis.

During the study period, there were changes in the SUS’s incorporation of technologies, beginning with the enactment of Law 12,401/2011 for the creation of the National Commission for the Incorporation of Technologies (Comissão Nacional de Incorporação de Tecnologias - Conitec) [15], which reorients the way of incorporating new technologies into SUS.

Through Conitec, the evidence-based effectiveness, accuracy, efficacy, and safety of the integrated technologies are analysed and the economic benefits and costs of these technologies are compared with those of technologies already incorporated [15]. Using this strategy, the process of analysis and incorporation of new technologies into SUS has become more transparent, responsive, and democratic [14]. Despite these changes, the number of lawsuits continues to increase in Brazil.

Accordingly, studies on the judicialization of health should be conducted to understand both this phenomenon and the SUS’s PA policy.

This study aimed to characterize medicines lawsuits filed between 2010 and 2014 against the SES/SP, following PA policies.

## Methods

This descriptive study used secondary data on medicines lawsuits filed against the SES/SP between 2010 and 2014. The data source was the computerized S-Codes system created and used by the SES/SP [12]. All of data was used in the study and there wasn’t missing.

After the judicial system decides a lawsuit in the claimant’s favour and issues an order, that order is forwarded to the SES/SP for compliance. The process includes an initial application prepared by the patient’s attorney, a copy of the patient’s personal documents, a description of the products/treatments requested, a medical report with the description of the patient’s disease, and a judicial order prepared by the judicial system. Next, the claim is registered in the S-Codes system by SES/SP technicians.

In this study, the S-Codes system generated a report that enabled the characterization of lawsuits and development of frequency measures in a manner that considered the number of lawsuits per year, the categories of the items claimed (medications, medical and hospital supplies, nutritional products, and medical and hospital procedures), the identity of the person who filed the lawsuit (lawyer, public defender, or prosecutor), type of lawsuit (normal proceeding, injunction, civil action, or other), the category of the prescriber (medical or otherwise), and the type of service that provided the prescription (private clinic, PHCU, unspecified hospital, teaching hospital, general hospital, specialist hospital, specialized clinic, outpatient clinic, or polyclinic). Data were analysed using the statistical software Stata, version 11.0, and Excel 2013.

The requested medicines were classified using Anatomical Therapeutic Classification (ATC) categories [16], considering the presence or absence of registration in the National Health Surveillance Agency (Agência Nacional de Vigilância Sanitária - Anvisa). This classification had already been input into the S-Codes database and enabled calculation of the frequency of these categories. The medicines were also grouped according to active principle, considering all similar dosage forms.

To assess whether medicines were incorporated into the SUS, during the study period, clinical protocols established by the Ministry of Health were used considering the items’ date of incorporation [4, 17, 18]. The consistency of the requests with the clinical protocols of the Ministry of Health—i.e., whether the requests could have been fulfilled by the PA programmes—was evaluated based on each patient’s diagnostic information.

### Statistical analysis

For the analysis of the number of lawsuits per lawyer, the number of lawyers responsible for 25%, 50%, 75%, and 100% of the lawsuits was calculated. The same procedure was done with the prescribers.

The Gini coefficients were calculated for the calculation of the number of lawsuits per lawyer and per prescriber. This coefficient was calculated using the Lorenz curve and corresponded to the area between the curve and the diagonal line. This coefficient varies between 0 and 1, where 0 corresponds to complete equality and 1 corresponds to complete inequality [19].

## Results

Between 2010 and 2014, 56,345 lawsuits were filed against the State Health Secretary of São Paulo to request medications, medical and hospital supplies, nutritional products, or medical and hospital procedures. The number of claims increased approximately 63% during the study period.

In 62.0% of the cases evaluated, the State of São Paulo was the sole defendant, and in 37.6% of the other cases, governance was shared with other public entities (federal, state of Sao Paulo, and municipal), as shown in Table 1.

**Table 1:** Description of lawsuit, São Paulo, 2010 and 2014

Variable	n	Prevalence % (IC 95%)
Responsibility to care of lawsuit (*n* = 56.345)		
State of São Paulo	34,952	62,0 (61,6–62,4)
State shared	21,207	37.6 (37,2–38,0)
Other	186	0,3 (0,2–0,3)
Categories of the items claimed (*n* = 148,236)		
Medications	91,931	62.0 (61,7–62,2)
Medical and hospital supplies	40,121	27.0 (26,8–27,2)
Nutritional products	8296	5,5 (5,4–5,7)
Medical and hospital procedures	6857	4,6 (4,5–4,7)
Other items	1031	0,6 (0,6–0,7)
Identify of the person who filed the lawsuit (*n* = 56,345)		
Lawyer	36,170	64.2 (63,7–64,5)
Public defender	7789	13.8 (13,5–14,1)
Prosecutor	5132	9.1 (8,8–9,3)
Uninformed	7254	12.8 (12,5–13,1)
Type of lawsuit (*n* = 56,345)		
Normal proceeding	34,649	61.4 (61,0–61,8)
Injunction	14,783	26.2 (25,8–26,6)
Civil action	4786	8,4 (8,2–8,7)
Other	2127	3,7 (3,3–3,9)
Category of the prescriber (*n* = 56,345)		
Medical	55,697	98,8 (98,7–98,9)
Otherwise	548	1,1 (1,0–1,2)
Type of service that the medical prescription was drawn up (*n* = 56,345)		
Private clinic	26,957	47,8 (47,4–48,2)
PHCU	10,673	18,9 (18,6–19,2)
Teaching hospital	7124	12.7 (12,3–12,9)
General hospital	4334	7.7 (7,6–7,7)
Specialized clinic/outpatient clinic/Outpatient clinic uninformed/Polyclinic	2378	4,2 (4,0–4,3)
Unspecified hospital	646	1.1 (1,0–1,2)
Specialist hospital	538	1.0 (0,8–1,0)
Uninformed	3695	6,5 (6,3–6,7)
Item claimed of medication included of components of the PA program (*n* = 91.931)		
Basic	15,265	16,6 (16,3–16,8)
Specialized	11,104	12,0 (11,8–12,2)
Strategic	346	0,3 (0,3–0,4)
No one	65,216	70,9 (70,6–71,2)

Source: S-Codes

With regard to the prescriptions in the claims, 62.0% corresponded to medications, and 38.0% corresponded to other items, including medical and hospital supplies (27.0% of cases), nutritional products (5.5% of cases), medical and hospital procedures (4.6% of cases), and other items (0.7% of cases). It is noteworthy that a lawsuit can request more than one item. The data obtained in this study corresponded to 148,236 items (6935 different items), which were prescribed in 56,345 legal proceedings (Table 1).

The analysis of the distribution of lawsuits according to the identity of the filing party indicated that most (64.2%) of the lawsuits were filed by private lawyers. Defenders and prosecutors were responsible for 22,9% of the claims. Information identifying the filing party was not reported in 12.8% of cases. Ordinary lawsuits were the predominant type of lawsuit, representing 61.4% of cases, followed by injunctions (26.2% of cases) and other types of lawsuits (12.1% of cases) (Table 1).

With regard to the origin of the prescriptions at issue, 47.8% (26,957) were issued in private clinics, 22.5% (12,642) were issued in hospitals, 18.9% (10,673) originated in the PHCU, and 4.2% (2378) were issued in specialty clinics; in 6.5% (3695) of cases, the point of care could not be defined. Of the requests issued in hospitals, 56.3% (12.6% of the total, 7124 prescriptions) came from teaching hospitals (Table 1).

Medications corresponded to 62.0% (91,931) of the requests (Table 1). In 8.9% of cases, the court required medication from a specific manufacturer, and thus, generic or similar medicines available through the programme could not be dispensed to the patient.

The claimed medicines, 29.1% (26,715 items) could have been provided via the PA program components, which are 12.0% of specialized component, 0.3% of the strategic component, and 16.6% of the component program basic (Table 1).

The medicines were grouped according to their ATC categories [16]. Medicines that targeted the digestive system and metabolism, nervous system and cardiovascular system were the most requested and corresponded to 57,107 (62.6%) medications that were requested in lawsuits, 24,0% A – Alimentary Tract and Metabolismo, 21,3% N – Nervous System e 17,3 C – Cariovascular System.

The 91,931 requests for medications had 4614 different presentations. Of these medications, 3.5% (160) were not available on the Brazilian market. In this context, the registration and price of the medicines sold in Brazil must be approved by Anvisa.

An analysis of the medicines grouped according to their active ingredients and dosage forms indicated that of the 20 most requested medicines, insulin glargine (6.3%) and insulin aspart (3.3%) were the two most requested medications, followed by methylphenidate (3.1%) and ranibizumab (3.0%) (Table 2).

**Table 2:** Number of lawsuits involving the 20 most requested medicines, São Paulo, 2010–2014

Item	Number (%)
Insulin glargine - vial/cartridge/disposable pen	3539 (6.3)
Insulin aspart - vial/cartridge/disposable pen	1849 (3.3)
Methylphenidate - capsule/extended-release tablet	1773 (3.1)
Ranibizumab - ampoule	1710 (3.0)
Insulin lispro - vial/cartridge/disposable pen	1565 (2.8)
Acetylsalicylic acid (100–325 mg) - tablet/coated tablet/slow-release tablet	1309 (2.3)
Clopidogrel - tablet	1224 (2.2)
Levothyroxine (38–200 mcg) - tablet	966 (1.7)
Glucosamine + chondroitin - sachet/tablet	968 (1.7)
Cinacalcet hydrochloride - coated tablet	906 (1.6)
Boceprevir 200 mg - gelatine capsule	844 (1.5)
Simvastatin (5–80 mg) - tablet	842 (1.5)
Insulin detemir - cartridge/disposable pen	834 (1.5)
Omeprazole - tablet/capsule	799 (1.4)
Insulin glulisine - vial/cartridge/disposable pen	801 (1.4)
Sodium hyaluronate - injectable solution/vial	771 (1.4)
Pregabalin - capsule	761 (1.4)
Metformin + vildagliptin - capsule	733 (1.3)
Rosuvastatin (5–40 mg) - tablet	730 (1.3)
Zoledronic acid - injectable solution/vial	675 (1.2)
Subtotal:	23,559 (41.9)
TOTAL	56,345 (100)

Source: S-Codes

**Fig. 1:**
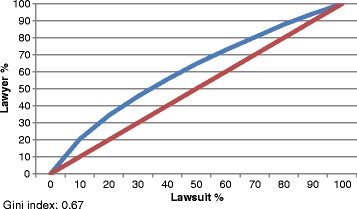
Lorenz curve. Number of lawsuits per lawyer. São Paulo, 2010–2014

**Fig. 2:**
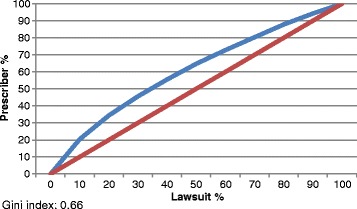
Lorenz curve. Number of lawsuits per prescriber. São Paulo, 2010–2014

We also assessed the possible predominance of lawyers and physicians in the lawsuits. Table 3, Figs. 1 and 2 show the percentage of lawyers and physicians represented in the lawsuits. An analysis of the distribution of all items (Figs. 1 and 2) and the 20 most-requested items (Table 3) indicated that this distribution was neither regular nor linear.

**Table 3:** Distribution of the 20 most requested medicines in lawsuits and concentration of lawyers and physicians, São Paulo, 2010–2014

Item	Lawyer - percentile (%)				Prescriber - percentile (%)			
	25%	50%	75%	100%	25%	50%	75%	100%
Insulin glargine - vial/cartridge/disposable pen	12.3	24.5	38.1	100	9.4	18.9	35.8	100
Insulin aspart - vial/cartridge/disposable pen	12.0	24.1	30.1	100	10.0	19.9	36.8	100
Methylphenidate - capsule/extended-release tablet	9.8	19.5	34.9	100	5.6	11.5	24.5	100
Ranibizumab - ampoule	14.1	28.3	42.4	100	5.6	11.4	23.5	100
Insulin lispro - vial/cartridge/disposable pen	12.5	24.9	38.6	100	11.6	23.3	42.0	100
Acetylsalicylic acid (100–325 mg) - tablet/coated tablet/slow-release tablet	13.3	26,4	41.2	100	14.1	28.2	44.6	100
Clopidogrel - tablet	15.7	31.5	47.2	100	15.5	14.7	46.6	100
Levothyroxine (38–200 mcg) - tablet	14.6	29.1	49.2	100	14.7	29.2	49.2	100
Glucosamine + chondroitin - sachet/tablet	16.7	33.5	50.2	100	23.3	45.2	73.9	100
Cinacalcet hydrochloride - coated tablet	14.2	29.3	43.9	100	7.3	14.6	31.6	100
Boceprevir 200 mg - gelatinous capsule	7.6	15.3	22.9	100	3.7	7.5	16.2	100
Simvastatin (5–80 mg)/tablet	15.0	30.8	46.8	100	16.5	33.1	49.6	100
Insulin detemir - cartridge/disposable pen	15.7	31.4	47.1	100	12.7	25.4	44.0	100
Omeprazole/tablet/capsule	24.9	49.7	75.1	100	19.9	39.9	59.7	100
Insulin glulisine - vial/cartridge/disposable pen	13.5	27.1	40.7	100	11.2	22.4	41.6	100
Sodium hyaluronate - injectable solution/vial	15.7	28.7	47.5	100	16.0	39.4	81.2	100
Pregabalin - capsule	14.7	29.5	44.5	100	14.6	29.0	46.1	100
Metformin + vildagliptin - capsule	15.9	31.9	48.4	100	12.7	25.5	41.8	100
Rosuvastatin (5–40 mg) - tablet	15.8	31.6	49.4	100	15.7	31.3	48.3	100
Zoledronic acid - injectable solution/vial	14.0	29.5	42.1	100	9.7	19.3	31.5	100

Source: S-Codes

The 56,345 claims had 11,865 lawyers and 15,069 different prescribers. Despite the large number of lawyers, only 20 (0.17%) of the lawyers filed 19.09% of the requests, whereas 20 (0.13%) of the medical prescribers were involved in 5.2% of the lawsuits (2927).

These results highlight the requests for boceprevir, methylphenidate, insulin glargine, and insulin aspart. For these items, 25% of the lawyers were responsible for filing more than 65% of the requests, and the same was true of medical prescriptions. For the other items, 25% of the lawyers were responsible for filing more than 50% of the requests, and the same was true of medical prescriptions, except for the requests for omeprazole and glucosamine + chondroitin, which did not follow this trend (Table 3).

Figure 1 shows that 50% of the lawsuits were filed by 64.2% of the lawyers. The same occurred with medical prescriptions, such that the prescriptions requested in 50% of the lawsuits were prepared by 64.7% of the prescribers (Fig. 2). The Gini coefficient was 0.67 and 0.66 respectively.

## Discussion

In Brazil, there has been much debate about the effects of the judicialization of health and its consequences both for the SUS and for the population. These studies often have different views of various social elements, which consider only one side of a multifaceted topic depending on their position in the political arena.

The judicialization of health has been implemented in countries in which health is a right. This process assures—through the vehicle of the courts—the guarantee of a constitutional right [20].

The significant increase in the number of medicines requests made via litigation has been attributed to the limitations of PA policies, the constraints and delay in the incorporation of new technologies into the SUS, and the population’s limited access to the medical treatments available from the SUS [21]. This increase can also be attributed to the judiciary’s interpretation of what constitutes comprehensive PA, as described in the 1988 Constitution, as the dispensation of all of the medications available in the Brazilian market without considering public policies, especially PA policies.

Because of this growing demand, studies have been conducted in various Brazilian states in an attempt to understand the judicialization process by tracing the profiles of lawsuits, making inferences about lawsuits’ claims, characterizing the various aspects involved in lawsuits, and suggesting alternative solutions [22].

We can summarize the potentially adverse effects of the judicialization of health as the compromise of the SUS’s principles, difficulties in PA management, and the careless use of medicines, which subjects claimants’ health to unnecessary risks [9, 23].

Previous studies have shown that judicial interference in health policies violates the principle of equality by favouring the demands of those who least need it at the expense of those who must rely exclusively on the public health system. As a result, existing inequalities are expanded [9, 23, 24]. Judicialization can lead to unequal access to the SUS because those who litigate have access to a broader array of health care services, whereas the rest of the population obtains only that which has been defined in the policies [9]. Another study has indicated a possible compromise of the principles of comprehensiveness because individual lawsuits are not extended to other holders of the same pathological conditions who could benefit from the object of the claims [23].

With regard to health management, judicialization of health generates a high degree of uncertainty for the public managers because of the uncontrolled increase in the number of lawsuits, the public resources needed to provide for the legal purchase of the medications, the impact of litigation on public accounts and the necessary cuts in other costs and policies [9, 23]. In addition, it generates an imbalance in the distribution of competencies within the system and often burdens the system’s most fragile element: the municipality [9].

In contrast, judicialization results in the judiciary’s negative interference in public health policies to ensure the constitutional right to health [25]. These lawsuits guarantee the Brazilian Constitution’s right to health. The use of the courts to request medicines available in PA programmes is a legitimate strategy to ensure the full exercise of the individual right to therapeutic care, which includes the right to health pursuant to Brazilian law [23].

Other authors defend the judicialization of public policies, particularly the policy for medication distribution, as a strategy to respond satisfactorily to new and increasing health demands [20]. In this context, judicialization would function as an instrument to assess individual and collective health needs [24].

The survey conducted in the SES/SP between 2010 and 2014 indicated an increase of approximately 63% in the number of lawsuits demanding the provision of health-related products, primarily medications. Although this increase follows an increase in the number of lawsuits at the federal level [14, 26], it has not been observed in other states such as Rio Grande do Sul [27].

Most of the items requested in the lawsuits under study are medicines, and in 8.9% of cases, the judiciary ordered the use of “brand-name medicines”, i.e., medications produced by a particular manufacturer; in other words, the government was forbidden to supply the patient with generic or similar products either purchased by the public administration or available in PA programmes. However, brand-name medicines dispensation does not comply with SUS regulations because these medicines should be prescribed based on the Brazilian Common Denomination, and their trade names cannot be used [28]. Therefore, a judicial order to purchase brand-name medicines violates Brazilian law.

Of the requested medications, 3.5% were not registered by Anvisa and therefore could not be marketed in Brazil or prescribed and dispensed under the SUS because the SUS is not allowed to dispense medicines without registration [15]. Drug lawsuits involving items not registered by Anvisa have previously been described [7, 12, 26, 29] (Table 4).

**Table 4:** Main characteristics of lawsuits, 2010 a 2014, SES/SP

Variable		Prevalence (%)	Importants obervations	Consequences
Categories of the items claimed	Medications	62,0	8.9% of cases, the court required medication from a specific manufacturer.	These demands did not follow the SUS regulations, and did not respect public policies, particularly PA policies.
			3.5% were not available on the Brazilian market.	These demands did not follow the SUS regulations, and did not respect public policies, particularly PA policies.
			30% of the medications involved in court proceedings were supplied via PA programmer.	These demands did not follow the SUS regulations, and did not respect public policies, particularly PA policies.
			Most demanded drugs: insulin glargine and insulin asparte, both have not been incorporated into the SUS because of its low efficacy and cost effectiveness.	These demands did not follow the SUS regulations, and did not respect public policies, particularly PA policies. The increase in the number of lawsuits, preceding the incorporation of the item into the SUS.
Type of service that the medical prescription was drawn up	Private clinic	47,8	The most of the prescriptions were derived from private clinics.	Doctors’ unfamiliarity with the SUS prescribing treatments (protocol), the lack of agreement between professionals and SUS protocols, or the SUS inability to quickly incorporate new health technologies.
Type of lawsuit	Normal proceeding	61,4		Demonstrating the individual nature of these claims.
Identify of the person who filed the lawsuit	Private lawyers	64,2		Demonstrating the individual nature of these claims, and this data denotes the socioeconomic level of the claimants.
Responsibility to care of lawsuit	State of São Paulo	62,0		Contrary to laws, norms and protocols of the SUS.
Gini index				
Lawsuits per lawyer.		0.67	Inequality in the distribution between the number of cases and lawyers and the number of lawsuits.	Concentration of lawyers and physicians in filing lawsuits. Evidence that there are groups of lawyers and prescribers “specialized” in these actions.
Lawsuits per prescriber.		0,66	Inequality in the distribution between the number of cases and prescribers and the number of lawsuits.	

Our data indicate that prescriptions do not follow the legal requirements for dispensing medications via the SUS. In this respect, there is space for efficiency and commitment to the public interest by regional and federal councils of medicine to prevent such behaviours by physicians.

With regard to the classification of medicines according to their presence in PA programmes, it was observed that approximately 30% of the drug items requested in court proceedings belonged to PA programmes (Table 4).

With regard to the basic component, the patient with a prescription can acquire the needed product in the PHCU of each municipality. The lawsuit is justified in the event of a lack of patient access to the item because of its unavailability in the PHCU. Another reason for the presence of a high number of medications in the basic component is the physician and/or patient’s lack of knowledge about the SUS’s dispensing of a given item.

In the case of medications present in the strategic component, the analysis of our data did not allow the confirmation of whether the items could have been dispensed by the PA programme. This programme has specific protocols and therefore requires information about the patients’ clinical status to assess whether they can be served by the program; however, the number of items requested in this component was small.

We evaluated whether the prescription of medications provided via the specialized component of PA complied with the CPTG and found that only 22.7% of these items were in compliance with this protocol and could be dispensed to the population via this programme. Therefore, the remaining drug items (81.3%) were prescribed in violation of Ministry of Health protocols (Table 4).

With regard to the specialized component, in the same period, the number of items provided by the Ministry of Health increased [30]. Similarly, SES/SP data indicated that the number of patients served by the programme in the State of São Paulo increased 51.8%, from 416,468 to 632,068 patients. Despite the expansion in coverage and increase in the number of medicines incorporated in this component, the number of lawsuits continues to increase.

It is important to remember that lawsuits can involve prescriptions composed of several items and these prescriptions can include medicines available in PA programmes; this explains the presence of these items in lawsuits, and in this case, they would be included in the lawsuit but would not be the focus of the process.

Lawsuits involving medicines available in SUS programmes can indicate these programmes’ malfunction. In other words, despite providing resources, these programmes can present practical problems in dispensing, including frequency of supply, limited access to the dispensing units, bureaucratic and other barriers, or prescription that violates the CPTG. These lawsuits can also indicate a delay or failure to incorporate the item into these programmes.

Requests for new and innovative technologies offered by the pharmaceutical industry pose a major challenge in the form of increasing costs to the public health system. These expensive technologies—effective or not—have been developed in Brazil in the name of the principle of universal coverage, comprehensive care policies, and specificity of the health care market, which sells a product of substantial monetary value both to life and to quality of life. Therefore, there is a frequent dispute between fulfilling an individual’s right and fulfilling a collective right [31].

Pharmaceutical innovations are not always the best options available for use in public policies, particularly when considering the concept of cost-effectiveness in the decision to incorporate new technologies. There is no doubt that lawsuits have been an important strategy to obtain access to the latest generation of medicines: they are essential to evaluate the evidence on which their prescription and use are based [21].

The visualization of the processes showed 1387 different diseases described in the medical reports, among which diseases of the digestive system and metabolism are the most frequent, followed by diseases of the nervous system and cardiovascular system. The change in the epidemiological profile of the Brazilian population related to aging and socioeconomic conditions may explain the prevalence of chronic diseases in the demands studied [32].

The claims evaluated included 4614 different medicines, which were grouped according to their active ingredient and analysed with respect to their incorporation into the SUS. Insulin glargine was the most requested medicine, corresponding to 6.3% of the claims, followed by insulin aspart, corresponding to 3.3% of the claims (Table 4).

Studies on lawsuits filed in others states of Brazil have found insulin glargine to be one of the most demanded medicines [22, 23]. To treat diabetes, the SUS provides oral hypoglycaemic agents such as metformin, glibenclamide, gliclazide, neutral protamine Hagedorn (NPH) human insulin, and regular human insulin [33].

With respect to insulins, in the studied period, Conitec recommended that the SUS not incorporate both the long-acting insulin analogues glargine and detemir for the treatment of type 1 and 2 diabetes mellitus or the fast-acting insulin analogues lispro, aspart, and glulisine for the treatment of type 1 diabetes mellitus. Studies did not prove the superiority of the treatment provided in the SUS; in addition, these insulin products are not cost-effective [34, 35].

The treatment of diabetes requested in the lawsuits studied has no scientific basis justifying the non-use of the insulin prescribed in the SUS. It is necessary to evaluate if the benefits derived from the analogous insulins justify the additional expenses with their supply [36]. Therefore, the most in-demand product has not been incorporated into the SUS because of its low efficacy and cost effectiveness. However, its purchase and dispensing occur by court order, distant from technical deliberations.

The third most requested drug, methylphenidate, is used to treat attention deficit hyperactivity disorder (ADHD). The use of this medication has increased considerably in Brazil, and federal data indicate that the sale of methylphenidate in pharmacies and private drugstores in Brazil more than doubled between 2009 and 2011 [37].

Despite the increase in the number of medical prescriptions and the high number of lawsuits involving methylphenidate, until July of 2016, neither the medical community, civil society, nor the drug’s manufacturer had requested its incorporation into SUS [17].

The fourth most demanded item was ranibizumab, which was approved by Anvisa for the treatment of age-related macular degeneration (AMD), diabetic macular oedema (DME), and retinal vein occlusion (RVO). In 2012, Conitec issued an unfavourable opinion on incorporating ranibizumab into the SUS for the treatment of AMD. In 2015, this product was evaluated for the treatment of DME; however, an unfavourable opinion was reached about its inclusion, and bevacizumab was approved as a cost-effective alternative [17].

With respect to the other items that were the most requested in lawsuits, boceprevir hydrochloride 200 mg and cinacalcet were incorporated into the SUS for the treatment of hepatitis in July 2012 and the treatment of hyperparathyroidism secondary to renal disease in patients subjected to dialysis in September 2015. Cinacalcet was incorporated after the data analysis period.

The SUS routinely provides acetylsalicylic acid, levothyroxine, simvastatin, and omeprazole via PA programmes. In these cases, judicial interference to dispense them to the population would not be necessary.

Clopidogrel, glucosamine + chondroitin, sodium hyaluronate, and pregabalin are not available in the SUS, and lacked of incorporation request to Conitec. The same applies to metformin + vildagliptin, rosuvastatin, and zoledronic acid, which have not been incorporated [17]. However, for the last three items, in the therapeutic indications for which they are routinely used, other medicines present in the PA programmes are regularly provided, including metformin, atorvastatin, and bisphosphonates.

It is clearly shown that the provisions of these lawsuits do not meet the principles of SUS. Of the 20 most prescribed items, only clopidogrel, glucosamine + chondroitin, sodium hyaluronate, and pregabalin are not present in the official list of the SUS.

The increase in the number of lawsuits, preceding the incorporation of the item into the SUS, can be a strategy by medical institutions, university hospitals, patients/population, and the pharmaceutical industry to pressure the SUS to incorporate these medicines into PA programmes.

Our results indicate that most of the prescriptions were derived from private clinics. Among hospital institutions, teaching hospitals supplied most of the prescriptions (Table 4).

The SUS imposes rules for the prescription and use of medicines; prescribers should satisfy the established clinical protocols. The fact that prescriptions from private clinics accounted for approximately 50% of all the claims might be attributable to these doctors’ unfamiliarity with prescribing treatments in the SUS, the lack of agreement between professionals and SUS protocols, or the inability of the SUS to quickly incorporate new health technologies.

Another issue that should be addressed is the influence of the pharmaceutical industry in medical prescriptions, with medical professionals being more vulnerable to this industry segment. Recent prohibitions and restrictions related to the advertising of new medicines have prompted the pharmaceutical industry to use alternative forms of advertising. Media and medical conferences, along with scientific studies, have represented excellent and efficient strategies to achieve the industry’s goals and to enable scientific information to reach the population [38].

Regarding the high number of comings of educational institutions prescriptions, that fact may indicate that the university hospitals by their nature of work, as clinical studies and research new technologies also exert pressure on incorporating PA programmes.

With regard to judicial data, most of the cases were ordinary, individual lawsuits filed by private lawyers, with the State of São Paulo named as the sole defendant. Studies on right-to-health lawsuits against the public authorities indicate that much of this demand focuses on individual claims for medications [22, 25] and the predominance of private lawyers advocating for claimants in the large states of Brazil [22].

Citizens’ strategy to ensure their rights using the judicial system occurs in two distinct dimensions. The first dimension represents the individual interests and includes both the public defender and private law firms. Their demands are related to the individual rights that should be guaranteed by the State and the purchase of products that are necessary for the maintenance or recovery of individuals’ health. The second dimension represents the collective interests, which are defended by civil society associations and the District Attorney’s Office and are aimed at guaranteeing specific groups of patients access to products and services [20].

Therefore, individual claims predominate in the State of São Paulo in a context in which health should be equitable and a right of all Brazilian citizens.

Considering the increasing number of lawsuits with the characteristics presented, we emphasize the individual nature of judicial decisions. The judiciary should consider litigants’ demands in light of two legal principles: the reserve for contingencies and proportionality.

The reserve for contingencies is a guarantee of a person’s legal rights, provided as the availability of relevant public resources [39]. The argument is that managers work with finite budgets; therefore, these budgets should be effectively used to better serve the population based on the public policies that have been implemented. This principle has been questioned in the public health sector because finite budgets can limit the fulfilment of the right to health, thus violating that right. In this case, the judge should evaluate the possible failure of the executive branch, and reasonableness and common sense should balance judicial decisions to ensure true justice [39].

The principle of proportionality is intended to balance individual rights with society’s aspirations. In this context, the right to health should be preserved by avoiding abuse of that right, i.e., providing maximum benefit to the community with minimal sacrifice from individual members. When two rights—the right to individual health and right to collective health—are evaluated, the possible solution is not the dichotomy of the right to health and the financial interests of the State, but by the need for judicial decisions are also based on technical aspects -scientific-based evidence [40].

The operation of the SUS is the joint responsibility of the Union, states, and municipalities; thus, any of these entities can be named as sole defendants in lawsuits filed to ensure access to medications. According to this logic, lawsuits are decided without consideration of agreements made between federal entities, potentially disrupting management in the Health Departments and Ministry of Health because these entities are required to execute services that do not belong to their scope of action.

The 56,345 cases had 11,865 lawyers and 15,069 prescribers. Despite the large number of lawyers, only 20 (0.17%) lawyers accounted for 19.1% of the claims filed against the SES/SP. In the case of physicians, only 20 (0.13%) prescribers, who appeared in most of the cases, corresponded to 5.2% of the lawsuits. These results suggest a predominance of lawyers and physicians involved in the lawsuits evaluated. The predominance of lawyers and physicians and the data on the Lorenz curves indicate a predominance of lawyers and prescribers.

The Gini coefficient of 0.67, corresponding to the number of lawsuits per lawyer, indicates inequalities in the distribution of the lawsuits among the lawyers and evidences the predominance of lawyers in the filing of lawsuits. The same tendency was observed among physicians (Gini coefficient of 0.66) (Table 4).

This study used secondary data obtained from the report prepared using S-Codes. Therefore, many questions could not be answered with the available data and should be addressed with studies that use other methodological designs capable of addressing different topics, including the potentially limited access to medications supplied via PA programmes, potential shortages of these medications, the prevention of lawsuits that demand medications supplied via the strategic components, and pressure from the medical class and pharmaceutical industry for the inclusion of medicines that do not meet the requirements of the policy of incorporation of new technologies of the SUS, among other issues.

However, one of the strengths of this study was that the number of lawsuits filed between 2010 and 2014 was evaluated in one of the most populous Brazilian states, representing more than 56,000 claims. To the best of our knowledge, no similar studies have evaluated such a large number of requests.

## Conclusion

The judicialization of health in the State of São Paulo with the characteristics presented herein is a threat to the SUS. Most of the lawsuits were individual, did not follow the SUS regulations, and did not respect public policies, particularly PA policies.

Although these circumstances are not simple, these lawsuits should be decided in a manner that guarantees the constitutional right to health without violating public policies, including PA policies.

The hypothesis that the number of lawsuits increased because of the limited incorporation of new medicines into the SUS is a misconception when we consider that despite the changes in the policy of technological incorporation within the SUS with the creation of Conitec [15], which made the analysis and incorporation of new technologies into the SUS more transparent, responsive, and democratic [14], the number of lawsuits continues to increase in Brazil. Therefore, the argument that the increase in the number of lawsuits is often attributed to the failed or delayed incorporation of new technologies into the SUS is unjustified.

## Appendix

**Table 5:** List of diseases and complications treated by the specialized component of PA. February 2015

Acne	Gaucher disease	Osteoporosis
Acquired pure red cell aplasia	Glaucoma	Paget’s disease - osteitis deformans
Acromegaly	Growth hormone deficiency - hypopituitarism	Parkinson’s disease
Acute coronary syndromes	Guillain-Barré syndrome	Phenylketonuria
Alzheimer’s disease	Haemangioma	Polycystic ovary syndrome and
Amyotrophic lateral sclerosis	Heart and lung transplantation	Primary adrenal insufficiency - Addison’s disease
Anaemia in chronic renal failure	Heart transplant	Primary immunodeficiency
Ankylosing spondylitis	Hereditary angioedema	Prophylaxis of reinfection by the Hepatitis B virus following liver transplantation
Aplastic anaemia	Hereditary ichthyosis	Psoriasis
Asthma	Hirsutism	Psoriatic arthritis
Autoimmune haemolytic anaemia	HIV disease resulting in other diseases	Pulmonary arterial hypertension
Autoimmune hepatitis	Hyperphosphatemia in chronic renal failure	Reactive arthritis
Bone marrow or pancreas transplantation	Hyperprolactinemia	Reiter’s disease
Central precocious puberty	Hypoparathyroidism	Renal osteodystrophy
Chronic obstructive pulmonary disease	Idiopathic thrombocytopenic purpura	Renal transplantation
Chronic viral hepatitis B	Inflammatory spondylopathy	Rheumatoid Arthritis
Congenital adrenal hyperplasia	Iron overload	Rickets and osteomalacia
Crohn’s disease	Leiomyoma of the uterus	Schizoaffective disorder
Cystic fibrosis	Liver transplantation	Schizophrenia
Dermatomyositis and polymyositis	Lung transplantation	Sickle cell disease
Diabetes insipidus	Lupus systemic erythematosus	Spasticity
dyslipidaemia	Multiple sclerosis	Systemic sclerosis
Endometriosis	Myasthenia gravis	Turner Syndrome
Epilepsy	Nephrotic syndrome	Ulcerative colitis
Exocrine pancreatic insufficiency	Neutropenia	Viral hepatitis C
Focal dystonia and hemifacial spasm	Non-infectious posterior uveitis	Wilson’s disease

Source: Ministry of Health homepage

## Abbreviations

ADHD: Attention deficit hyperactivity disorder
AMD: Age-related macular degeneration
Anvisa: National Health Surveillance Agency (Agência Nacional de Vigilância Sanitária
ATC: Anatomical Therapeutic Classification
Conitec: National Commission for the Incorporation of Technologies (Comissão Nacional de Incorporação de Tecnologias)
CPTG: Clinical protocols and therapeutic guidelines
DME: Diabetic macular edema
IJHSP: Judicialization of Health of Sao Paulo
NPH: Neutral protamine Hagedorn
PA: Pharmaceutical assistance
PHCU: Primary health care units
Rename: National List of Essencial Medicies (Relação Nacional de Medicamentos)
RHD: Regional Health Departments
RVO: Retinal vein occlusion
SES/SP: State Health Secretary of São Paulo (Secretaria de Estado de Saúde de São Paulo)
SUS: Brazilian Universal Health System (Sistema Único de Saúde)
